# Scoping review on the perceptions and attitude of women on methods for collecting cervicovaginal samples for Human Papillomavirus testing in Sub-Saharan Africa

**DOI:** 10.1371/journal.pgph.0004641

**Published:** 2025-05-23

**Authors:** Uduak Ima Andrew-Bassey, Deborah Olamiposi Oke, Michael A. Okunlola, Imran Morhason-Bello

**Affiliations:** 1 Pan African University Life and Earth Sciences Institute (Including Health and Agriculture), University of Ibadan, Ibadan, Nigeria; 2 Centre for Medical Informatics and Professional Development, Ibadan, Nigeria; 3 Department of Epidemiology and Medical Statistics, Faculty of Public Health, University of Ibadan, Ibadan, Nigeria; 4 HPV Consortium, College of Medicine, University of Ibadan, Ibadan, Nigeria; 5 Obstetrics and Gynaecology Department, Faculty of Clinical Sciences, College of Medicine, University of Ibadan, Ibadan, Nigeria; 6 Institute for Advanced Medical Research and Training, College of Medicine, University of Ibadan, Ibadan, Nigeria; Purdue University, UNITED STATES OF AMERICA

## Abstract

The burden of cervical cancer (CC) continues to rise in Sub-Saharan Africa (SSA) while some high-income countries are approaching elimination targets. Self-sampling for Human Papillomavirus (HPV) test for CC screening is increasingly used globally to accelerate wide coverage but some have reported barriers against its use. This scoping review explored published literature on the perception and attitude of women on the methods for collecting cervicovaginal samples for HPV testing for CC in SSA. This involves a review of electronic databases including Pubmed, Cochrane, Google Scholar, and African Journal Online. The review was limited to published English articles between 2013–2023 using the Arksey and O’Malley framework. Included studies were articles that used perception, attitude, perspective, or acceptability as primary or secondary outcome variables. Of the 137 articles, 131 articles were excluded due to duplicates and ineligibility. Six studies reported that women perceived self-sampling to provide better privacy and comfort, five studies reported that self-sampling was an easier procedure, five studies reported self-sampling was less painful, four reported that self-samples caused lesser embarrassment, three studies reported that women were willing to self-sample, and five studies reported it to be associated with better confidentiality than clinician sampling. Six studies reported that women perceived that the biological samples collected by the clinicians were more reliable compared to self-collected samples. Three studies showed that women preferred self-sampling in a private place in the hospital because they can seek reassurance from clinicians, and reduce the risk of financial burden associated with multiple visitations to the hospital. This shows that self-sampling is preferred relative to clinician-initiated collection of samples for HPV-based CC screening. It is important to emphasize increased sensitization on the reliability of self-sampling before asking the women to self-collect cervicovaginal samples.

## Introduction

The primary cause of almost all CCs is the persistence of high-risk human papillomavirus (HPV) infections in the transformation zone for nearly 2–7 years [1–3]. The latency period between the acquisition of high-risk HPV infections, its persistency, and the development of precancer and invasive cancer provides an opportunity for secondary prevention strategy including cytology and HPV-based testing [4]. Studies showed that the proportion of women who screen for CC is relatively low [5]. For example, a study reported that only 3.5% of women aged 25–65 years had ever screened for CC in the last three years [6]. Women in low-middle-income countries (LMICs) often face the challenges of poor access to screening services, lack of awareness and knowledge on cervical cancer (CC), screening, and sociocultural influences that prevent them from screening [7–9].

Some of the barriers associated with low CC screening using the clinician sample collection method include inadequately trained personnel, lack of functional referral and laboratory facilities, and lack of transport and follow-up systems [9,10]. Other barriers include embarrassment/shyness, pain, and/or dislike associated with pelvic examination, and spousal disapproval [11]. The World Health Organization (WHO) recommends HPV testing and treatment for women at risk of developing CC as part of efforts to eliminate the disease globally [12]. Self-sampling (SS) of cervicovaginal samples presents a unique opportunity to mitigate the challenges of barriers associated with healthcare provider’s collection of samples for HPV testing and cytological screening [13,14]. HPV testing presents women with the option of self-collection of cervicovaginal samples by removing the barriers associated with a third person’s engagement to collect cytology samples.

Studies have demonstrated that the quality of biological samples collected and DNA extracted from SS are similar to those collected by healthcare providers (HCP) [15–18]. Despite the opportunities that SS presents, it is important to assess the perception of women within the African context as a preliminary step and its implication in integrating this method of sample collection for CC screening programs. The perception of women who had experienced SS in SSA is yet to be reviewed across different settings to fully understand their preferences and concerns. This scoping review explored published literature on perceptions and attitudes of women on the collection of cervicovaginal samples for HPV testing in SSA, and it also identified potential gaps with this method as a proxy for assessing the effectiveness of CC screening in SSA.

### Objective

The objective of this review is to explore published literature on the perception and attitude of women on the methods for collecting cervicovaginal samples for HPV testing for CC in SSA.

## Methods

### Protocol and registration

The protocol for this review has been published (https://doi.org/10.1136/bmjopen-2024-085408) [19].

### Eligibility criteria

The eligibility requirements were created using the PICO format (population, intervention, comparator, and outcome) (Table 1). The studies were qualitative, quantitative, or mixed-method designs (a) published in peer-reviewed journals as either observational or experimental designs on the perception or attitude of women on methods of collecting cervicovaginal samples for CC screening and, (b) data collected in SSA among indigenous black women population. We excluded case reports, letters to editors, or expert opinions without primary data on methods of collecting samples for CC screening and original articles on this subject that were conducted outside SSA. Only English publications were included to avoid the cost and time required to translate foreign languages.

**Table 1:** Table showing eligibility criteria for the review using PICO format.

Categories	Inclusion Criteria	Exclusion Criteria
**Participants**	Women between the ages 18-65 years that have been previously screened for cervical cancer	Transgendered women without a cervix, and women that have never screened for cervical cancer
**Intervention**	Cervical cancer screening with samples collected either by oneself or by a health care provider or both	Samples collected from the cervix for other purposes other than cervical cancer screening
**Comparator**	Self-sampling; Provider sampling	Provider sampling only; Self-sampling only
**Outcome**	Perception, attitude, perspective, and/or acceptability	-

### Information sources

A systematic search of electronic databases including PubMed (a medical/health-related journal), Cochrane (Cochrane evidence provides high-quality information to enhance your healthcare knowledge and decision-making), AJOL (African Journals Online is rich in African journals), and Google Scholar (indexes the full text or metadata of scholarly literature across an array of publishing formats and disciplines) was conducted. In addition, grey literatures were reviewed to complement the original search strategy. The search criteria were limited to published original research studies conducted in community or clinical settings in SSA in the last ten (10) years (2013–2023).

### Search

The following keywords were used to search the databases: HPV/ Human papillomavirus/ papillomavirus, cervical cancer/ adenocarcinoma, sample collection, sample taking, self-sample, cervical cancer screening, perception/view, attitude, and SSA using sub-regions within SSA (West Africa OR East Africa OR Central Africa OR Southern Africa), and by specific country names. Boolean terms AND/OR were used to separate the keywords during the search. Medical Subject Headings (MeSH) terms were used in the search terms. The references and bibliographies of relevant articles were manually searched. The search was conducted between December 2023 and March 2024 including articles with publication dates in 2013–2023 (S1 Data).

### Selection of sources of evidence

The search was initially conducted by UAB verified by the two senior authors on 20^th^ March 2024 and finalized on 28th March 2024. The titles were imported into EndNOTE software to compile results from all the databases. UAB and DOO independently screened the articles/titles using PICO criteria for eligibility while IMB served as the tiebreaker. After, UAB and DOO screened all full texts of selected articles for eligibility and those found to meet the criteria were included for data extraction and mapping (S2 Data).

### Data charting process

**Fig 1:**
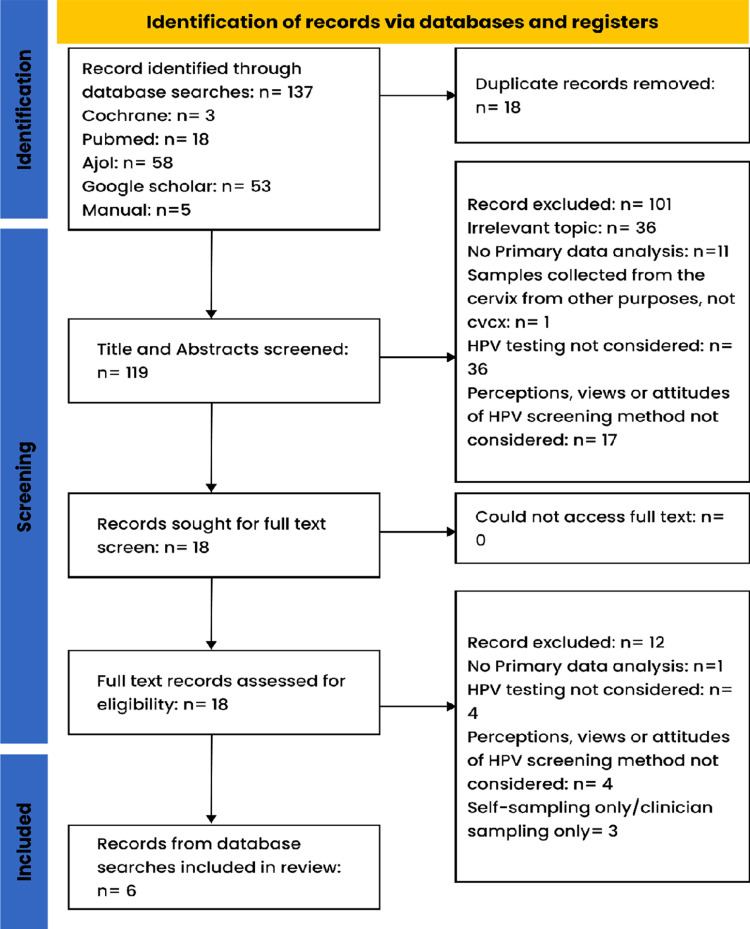
Preferred Reporting Items for Systematic Reviews and Meta-Analyses-extension (PRISMA) Flow chart diagram for scoping review.

Eligible studies were presented on a Preferred Reporting Items for Systematic Reviews and Meta-Analyses-extension (PRISMA) Flow chart diagram for scoping review (Fig 1) to summarise the process and number of articles that were finally selected for chart abstraction [20,21].

### Data items and synthesis of results

We created a two-data abstraction form with Microsoft Excel sheets using the following data charting fields. Sheet 1 includes data on authors, date, country, study population/ sample size, method of recruitment, type of cervical screening, and procedure for sample collection (Table 2). Sheet 2 includes data on authors, date, country, aim/objectives, measurement of perception (quantitative/qualitative/mixed), assessment for perception (descriptive/multivariate/test of association), methods of cervicovaginal sample collection conducted, outcome measure, and summary of key findings (Table 3). The data extracted were presented in Tables 2–3 to summarise eligible articles, description of perceptions and attitudes towards CC screening, and methods of collecting cervicovaginal samples.

**Table 2:** Description of included studies, method of participant’s recruitment, and type of cervical screening conducted.

S/N	Author (s)/Year/Country	Study Population/ Sample Size	Method of Recruitment	Type of Screening	Procedure for sample collection
1	^1^Saidu et al.	Women ages 30-65 years/822 questionnaires administered and 41 FGD’s	Women referred to a colposcopy clinic and a primary care site in Cape Town, SA were enrolled consecutively from February 2015 to May 2016. Equal numbers of HIV-positive and HIV-negative women were recruited.	Self-sampling (SS) for HPV testing followed by VIA and Colposcopy, appropriate histology specimens by a doctor.	All the participants self-collected a vaginal sample in a private room following a verbal explanation on how to collect the sample by a community health worker (CHW).
2	^2^Sormani et al.	Women 30-49 years/ 2201	Women referred to a colposcopy clinic and a primary care site in Cape Town, SA, from February 2015 to May 2016. Equal numbers of HIV-positive and HIV-negative women were recruited. No account for non-response rate.	SS for HPV testing, VIA & VILI conducted for positives, then treated by thermal ablation or loop electrosurgical excision of the transformation zone (LEETZ)	Women received instructions and a support guide that provided detailed visual information about the procedure.
3	^3^Obiri-Yeboah et al.	Women aged 18 years and above/194	Women attending the general medical outpatient and HIV clinics were recruited. A systematic random sampling of every fifth woman were selected from the list of daily attendants	SS for HPV testing and HCP samples for HPV testing	Recruited participants at the clinic were instructed on how to obtain Self collect vaginal samples using the careHPV brush and transport medium. Speculum examination was also conducted by the using a similar brush and transport media.
4	^4^Kohler et al.	WLWH aged 25 years and above/ 104	WLWH attending an HIV clinic for routine healthcare in Gaborone were recruited between March and April 2017.	SS for HPV testing and speculum examination by a clinician afterwards	Verbal description of how to use SS kit and distributed pictorial instructions. The cephid patient-collected vaginal swab instructions were adapted.
5	^5^Bakiewicz et al.	Women aged 25–60 years attended a patient-initiated screening/21	A non-random (purposive) sampling technique was used	SS for HPV, HCP sampling, gynaecologic examination and VIA.	Written instruction guide with illustrations of how to collect the self-sample as well as an oral instruction provided by a nurse to collect self-sample
6	^6^Berner et al.	Women aged 25-65 years/ 243	Recruitment of women attending routine cervical screening through convenience sampling	SS for HPV testing, HCP sampling and cytology	Written and oral instructions were given to self-sample unsupervised

^1^Saidu et al. 2019/ South Africa,

^2^Sormani et al. 2021/Cameroon,

^3^Obiri-Yeboah et al. 2017/ Ghana, SS, Self-Sampling; HCP, Healthcare Provider,

^4^Kohler et al. 2019/Botswana,

^5^Bakiewicz et al., 2020/Tanzania,

^6^Berner et al. 2013/ Cameroon; SS, Self-Sampling; HCP, Healthcare Provider

**Table 3:** Selected studies on perception and attitude of self-sampling in sub-Saharan Africa reporting key findings.

S/N	Author (s)/Year/Country	Aim: Perception/Attitude	Measurement for Perception (Quantitative/Qualitative/Mixed)	Assessment for Perception (Descriptive/Multivariate/ Test of Association)	Outcome Measure	Key Findings
1	^1^Saidu et al.	To explore women’s perceptions and acceptance of self-collection of samples for cervical screening and their willingness to do so, in a low-resource setting in South Africa (SA).	Mixed: quantitative assessment with a Likert scale and qualitative from the FGD. The questionnaire was self-administered	Mean/SD/ p-value; thematic analysis for FDG	Perceptions and beliefs surrounding SS	Positive attitude to SS was 93.6%. Positive perception to SS was 89.4%. FGD participants found SS easier, more comfortable, and less embarrassing than HCP sampling. Willingness to SS but expressed concerns regarding the quality of the specimen and the financial implications of returning to the clinic with it.
2	^2^Sormani et al.	To assess factors associated with women’s preferences related to self-HPV and perceptions around Self-HPV	Quantitative data using interviewer-administered questionnaire	mean(SD), frequency(percentage), chi-square, logistic regression	preference for clinician-sampled versus self-HPV	Comfortability was reported in 98.3% for SS and 96.9% during clinician sampling. Confidence: 99.3% SS vs 98.6% clinician sampling. Clinician expertise made women to rely more on HCP method compared to SS (76.1%).
3	^3^Obiri-Yeboah et al.	To determine the acceptability, feasibility and performance of alternative self-collected vaginal samples for HPV detection among Ghanaian women.	Quantitative data with interviewer-administered questionnaire	Frequency/Percentage, kappa for concordance	Acceptability, concordance	76.3% easier felt SS was than in 77.9% for HCP sampling. 57.7% would prefer SC over HCP sampling and 61.9% felt SC would increase their likelihood to access cervical cancer screening
4	^4^Kohler et al.	To assess the acceptability and preferences of HPV screening with SS and mobile phone results delivery among women living with HIV.	Quantitative data with interviewer-administered questionnaire	Frequency/Percentage, chi-square test	Acceptability of SS	90% found SS to be easy and comfortable. 95% expressed willingness for SS again (95%), 19% preferred SS over HCP sampling. A high number trust their clinicians & had low confidence in being able to sample correctly.
5	^5^Bakiewicz et al.	To investigate the feasibility and acceptability of HPV SS among Tanzanian women who attended a patient-initiated cc screening compared to provider-based HPV sampling.	Qualitative data with IDI	Thematic analysis	Feasibility and acceptability	Most women perceived SS as easy and comfortable though few experienced bleeding and pain.
6	^6^Berner et al.	To assess acceptability and preference for self-collected HPV tests compared with traditional physician-sampled Pap tests in a low-resource country	Quantitative data using a self-administered questionnaire	mean (SD), frequency (percentage), Mann-Whitney U-test, McNemar, binomial, chi-square, multivariate logistic regression	Acceptability and preference of SS over physician sampling	The acceptability score for self-HPV was 9.20 versus 11.80 for physician sampling (p<0.001). Preference was lower for self-HPV than physician sampling (29% vs 62%; p<0.001).

^1^Saidu et al. 2019/ South Africa,

^2^Sormani et al. 2021/Cameroon,

^3^Obiri-Yeboah et al. 2017/ Ghana,

^4^Kohler et al. 2019/Botswana; SS, Self-Sampling; HCP, Healthcare Provider,

^5^Bakiewicz et al., 2020/Tanzania,

^6^Berner et al. 2013/ Cameroon; SS, Self-Sampling; HCP, Healthcare Provider

## Results

### Selection of sources of evidence

One hundred and thirty-seven (137) articles were reviewed, six (6) met the inclusion criteria [1,16,18,22–24]. The flow chart for screening and reasons for exclusion were shown in (Fig 1).

### Characteristics of sources of evidence

Each included source of evidence has been charted on (Fig 1). Two (2) of these manuscripts were from Southern Africa (Botswana [23] and South Africa [22]); two (2) from Central Africa (Cameroon (2) [1,24]); One (1) from West Africa (Ghana) [16] and one from East Africa (Tanzania) [18]. Five (5) of eligible articles were conducted over five years ago (2017–2022) [1,16,18,22,23] and only one (1) study was conducted over ten (10) years ago [24]. The age range of participants were between 18 and 65 years old. Three (3) studies included women 25 years and above [18,23,24], two (2) included women 30 and above [1,22], and one (1) 18 and above [16]. All six (6) studies were hospital-based [1,16,18,22–24]. Of the four (4) studies that conducted quantitative analyses, two (2) studies were face-to-face interviews [16,23], while two (2) studies used self-administered questionnaires to collect data [22,24].

### Synthesis of results

#### Quality of DNA sample collected.

Six (6) studies assessed the quality of DNA samples [1,16,18,22–24]. Overall, women felt that the quality of DNA collected by HCP was more reliable compared to those collected during SS [1,16,18,22–24]. Some participants (76.1%) [1] and (23.1%) [23] believed that the quality of DNA collected by HCP was better because they were regarded as experts [1,23]. In a South African study, 45.1% women felt that the HCP could detect abnormalities aside from precancerous lesions [22]. A qualitative study conducted in Tanzania reported that most women perceived they were capable of collecting accurate samples in the presence of a nurse [18]. However, two studies reported a similar DNA quality in the cervicovaginal samples collected by HCPs and during SS [16,18]. Lastly, a study reported a sensitivity of 92.6% sensitivity and a specificity of 95.9% [16].

#### Perception and attitude of women about self-sampling.

Perception, attitude, perspective, and acceptability were used interchangeably in many studies [8,16,18,19,22–24]. The themes used to describe perception and attitude include privacy and comfortability; willingness to self-sample; pain experienced; embarrassment; anxiety; and confidence [8,16,18,19,22–24].

**Privacy and comfortability:** Five (5) studies reported that women perceived SS as a form of protection for their privacy and had better comfort when compared to clinician sampling methods [1,18,22–24]. A study reported that 16.1% of women preferred SS over HCP sampling due to the possibility of increased privacy [1]. In another study conducted among women living with HIV (WLWH) in Botswana, 90% of the women agreed that SS was more comfortable than HCP sampling [23].

**Willingness to self-sample:** Three (3) studies reported the willingness of women to self-sample [1,22,23]. A study conducted in South Africa reported that 93.9% women were willing to self-sample [22]. In a comparative study in Cameroon, 76.9% women willingness to self-sample compared to 23.1% women willing to present for provider sampling [1]. The same study reported that 99% of women were willing to self-sample again and refer other women for similar method of sample collection [1]. This is similar to findings from another study that reported 95% of women willing to self-sample again [23].

**Pain and anxiety:** Five (5) studies assessed the level of pain experienced during SS compared with provider sampling [1,18,22–24]. Overall, the majority of the studies reported that women perceived that SS was less painful relative to provider sampling [18,22–24]. A study reported that SS was associated with lesser pain while more than a third had a negative painful experience (37.5%) and physical discomfort (36%) [23]. Also, majority of women in two studies felt less anxious about SS 73% [24] and 96.5% [1]. However, 2 out of 11 women in a Tanzanian study reported per vaginam after SS [18]. Experience of bleeding was not reported during and after provider sampling.

**Embarrassment:** Four (4) studies assessed embarrassment as a measure of women’s perception [1,22–24]. A study in South Africa reported that 93.6% of women felt less embarrassed during SS compared with 88.2% of women during provider sampling [22]. Another study conducted in Cameroon reported that 99.3% of women felt less embarrassed during SS compared with 98.6% of women during provider sampling [1]. Also, another study in Cameroon reported that 84% of women felt less embarrassed to self-sample compared with the clinician sample [24]. These findings were corroborated by the FDG participants stating that they felt shy to open up to a clinician [22]. However, another study reported that an equal proportion of women felt embarrassed during SS and provider sampling (3%) [23].

**Confidence:** Five (5) studies assessed confidence as a measure of women’s perception [1,18,23,24]. A study conducted in Cameroon reported that 57.6% of women felt moderate or high confidence when they SS [24]. This is similar to a study among WLWH in Botswana which reported that 90.3% of women felt somewhat or extremely confident while SS [23]. A study in Cameroon reported a slightly greater proportion of women (SS: 98.9%; HCP: 99.4%) confident for provider sampling compared to SS [1]. However, a study conducted among Tanzanian women reported that the participants were not confident to self-sample except in the presence of a nurse [18]. Also, women were more confident when the sample was taken by a clinician (64.7%) than SS (59.5%) [22].

#### Procedure for self-sampling.

Five (5) studies reported women’s perception of SS collection procedures [16,18,22–24]. Overall, 76.3% [16], 82% [24], and 89% [23] women reported that SS was easy compared to provider sampling. The women were also satisfied with the content of the instructions given for SS [22,24]. A study conducted in Tanzania showed that women were more comfortable with SS when nurses or other healthcare workers were present than when they were left alone to self-collect [18].

#### Location for self-sampling for CC screening.

Three (3) studies reported preference for collecting cervicovaginal samples in the hospital rather than at home [1,18,22]. A study conducted in Cameroon reported that 96.6% of women preferred SS in a medical center over home-based screening [1]. This was because 36% of women were scared of performing the test inappropriately and 12% were scared of contamination if performed at home [1]. Other studies reported reassurance by clinicians [18,22], and the financial burden associated with visiting the health facility twice [22] as reasons women preferred SS in the hospital. However, a study conducted in Cameroon reported that 39% of women agreed to SS at home if introduced to regular screening [24].

## Discussion

### Summary of evidence

This scoping provides a synthesis of information on the perception and attitude of women towards sampling methods for CC screening. Our results confirmed the paucity of research on the subject in SSA. All the six studies were hospital-based and they described perception and attitudes using the following themes: Privacy and comfortability; Willingness to SS; Pain; Anxiety; Embarrassment and Confidence [1,16,18,22–24].

Self-sampling method was generally reported to protect their privacy and provided a better comfort than HCP-initiated sampling of cervicovaginal samples [1,18,22–24]. Privacy protection and comfort are important drivers that motivate clients to consent for gynaecological procedures particularly when it involves exposure of genital areas [25,26]. The presence of HCPs sometimes might create tension, anxiety, and feelings of intrusion when clients have not had any encounter or speak the same language with them [27,28]. It is also plausible that women could have mentioned privacy because of their feeling of being in control of themselves while collecting their samples. To mitigate this challenge, several strategies were employed including enforcement of using chaperons, counseling sessions before the procedure/examination, and use of audiovisual aids [29,30]. In other settings, women expressed similar reasons for privacy and comfort as a motivation for preferring self-sampling to provider-initiated sampling [14,31,32].

Three (3) studies showed that women were willing to self-sample [1,22,23]. Studies have shown that SS for HPV testing promises to increase screening rates because more women will be willing to self-sample compared to the HCP method [33–36]. However, 23.1% of women in one of the studies were willing to undergo provider sampling [1]. This could be a result of the confidence people/patients have in their HCPs [37].

Another important motivation for preferring SS is the perception by women that it is less painful than HCP-initiated sampling. Although none of the studies objectively assessed pain perception between SS and HCP-initiated sampling, the feeling of having lesser pain could be due to the general feeling of self-control during sample collection. It is also possible that the bleeding and pain experienced by participants could be suggestive that the screening instruction was not properly communicated to the study participants. Several other studies have reported similar findings regarding less pain during SS compared to more pain during provider sampling [31,33,38,39]. However, the bleeding experienced by WLWH could be attributed to the strong trust that exists between WLWH and their provider [40]. Hence, these women were nervous to self-collect samples. We recommend educational interventions tailored to providers to educate and encourage patients on the importance of SS options, especially providers working with WLWH.

Four studies reported that most of the participants believed that self-sampling made them feel less embarrassed compared to HCP sampling [1,22–24]. It is common knowledge that women had always expressed concerns such as feelings of embarrassment during any clinical scenarios that involve the insertion of instrument or examination of their vagina or cervix [41]. The incentive of allowing women to collect their samples may obviate this feeling of embarrassment including the extreme situation of vaginismus or frigidity.

The confidence to self-collect their samples for CC screening was overwhelmingly reported in four studies that were included in this review [1,18,23,24]. Some studies in this review showed that a few women that had secondary medical conditions such as HIV infections preferred health provider-initiated sampling relative to SS [1,22]. These women were not confident to perform SS and would rather want clinicians to examine them in addition to collecting their cervicovaginal samples. However, some women might require the assistance of healthcare providers to reinforce their confidence to perform SS [1,23,24]. Some studies outside SSA have reported similar findings that women with immunocompromised medical conditions have low self-confidence [42–44].

Overall, most of the women in the six studies perceived that the quality of DNA samples collected by the HCPs was more reliable compared to those self-collected [1,18,22–24]. This finding contrasts with findings from studies that tested the quality of DNA between specimen collected from SS and those collected by HCP [16,18]. For example, a study showed that the cervicovaginal samples collected by SS and HCP had similar high sensitivity (92.6%) and specificity (95.9%) [16].

Another important benefit of SS mentioned by women is the relatively lower overall cost of using this technique than the HCP-initiated sampling. For example, the cost of consumables for SS is generally lower and there might not be any need for hospital visitation [22,45,46].

### Limitations

The interpretation of findings from this scoping review is limited because of the following reasons. First, this scoping review did not include published manuscripts from non-English journals, and this omission could have introduced some bias. Second, out of the eligible studies, there was no community-based study which might have implication on the generalisability of our findings. Third, we did not assess for risk of bias in this review as it was not a systematic review. Despite these limitations, this review provided the first synthesis of evidence to compare SS and HCP initiated collection of cervicovaginal samples.

### Conclusion

In conclusion, this review showed an increasing preference for SS for collecting cervicovaginal sample for CC screening relative to HCP methods by women in SSA. It is important that clinicians and program planners increased sensitization of SS and encourage women to self-collect cervicovaginal samples as a motivation for increasing the uptake of CC screening in SSA. Future studies should explore motivations and barriers as well as possible integration of SS into different health system involved in CC screening.

## Supporting information

S1 Table Table showing eligibility criteria for the review using PICO format. (DOCX)

S2 Table Description of included studies, method of participant’s recruitment, and type of cervical screening conducted. (DOCX)

S3 Table Selected studies on perception and attitude of self-sampling in sub-saharan africa reporting key findings. (DOCX)

S1 Data Details of search strategy. (DOCX)

S2 Data Result from different databases. (XLSX)

S1 Checklist Preferred reporting items for systematic reviews and meta-analyses extension for scoping reviews (PRISMA-ScR) checklist. (DOCX)

## References

[1] Sormani J, Kenfack B, Wisniak A, Moukam Datchoua A, Lemoupa Makajio S, Schmidt NC, et al. Exploring factors associated with patients who prefer clinician-sampling to HPV self-sampling: a study conducted in a low-resource setting. Int J Environ Res Public Health. 2021;19(1):54. doi: 10.3390/ijerph19010054 35010314 PMC8744711

[2] Louie KS, de Sanjose S, Mayaud P. Epidemiology and prevention of human papillomavirus and cervical cancer in sub-Saharan Africa: a comprehensive review. Trop Med Int Health. 2009;14(10):1287–302. doi: 10.1111/j.1365-3156.2009.02372.x 19772550

[3] Aerssens A, Claeys P, Beerens E, Garcia A, Weyers S, Van Renterghem L, et al. Prediction of recurrent disease by cytology and HPV testing after treatment of cervical intraepithelial neoplasia. Cytopathology. 2009;20(1):27–35. doi: 10.1111/j.1365-2303.2008.00567.x 18510550

[4] Castle PE, Einstein MH, Sahasrabuddhe VV. Cervical cancer prevention and control in women living with human immunodeficiency virus. CA Cancer J Clin. 2021;71(6):505–26. doi: 10.3322/caac.21696 34499351 PMC10054840

[5] Yang L, Boily M-C, Rönn MM, Obiri-Yeboah D, Morhason-Bello I, Meda N, et al. Regional and country-level trends in cervical cancer screening coverage in sub-Saharan Africa: a systematic analysis of population-based surveys (2000-2020). PLoS Med. 2023;20(1):e1004143. doi: 10.1371/journal.pmed.1004143 36634119 PMC9882915

[6] Bruni L, Serrano B, Roura E, Alemany L, Cowan M, Herrero R, et al. Cervical cancer screening programmes and age-specific coverage estimates for 202 countries and territories worldwide: a review and synthetic analysis. Lancet Glob Health. 2022;10(8):e1115–27. doi: 10.1016/S2214-109X(22)00241-8 35839811 PMC9296658

[7] Denny L, Quinn M, Sankaranarayanan R. Chapter 8: screening for cervical cancer in developing countries. Vaccine. 2006;24 Suppl 3:S3/71-7. doi: 10.1016/j.vaccine.2006.05.121 16950020

[8] Oketch SY, Kwena Z, Choi Y, Adewumi K, Moghadassi M, Bukusi EA, et al. Perspectives of women participating in a cervical cancer screening campaign with community-based HPV self-sampling in rural western Kenya: a qualitative study. BMC Womens Health. 2019;19(1):75. doi: 10.1186/s12905-019-0778-2 31196175 PMC6567898

[9] Mantula F, Toefy Y, Sewram V. Barriers to cervical cancer screening in Africa: a systematic review. BMC Public Health. 2024;24(1):525. doi: 10.1186/s12889-024-17842-1 38378542 PMC10877795

[10] Ndejjo R, Mukama T, Musabyimana A, Musoke D. Uptake of cervical cancer screening and associated factors among women in rural uganda: a cross sectional study. PLoS One. 2016;11(2):e0149696. doi: 10.1371/journal.pone.0149696 26894270 PMC4760951

[11] Eche MT, Vermaak K. Knowledge, attitude and practice of female university students regarding human papillomavirus and self-sampling in KwaZulu-Natal, South Africa: a cross-sectional survey. BMC Womens Health. 2022;22(1):58. doi: 10.1186/s12905-022-01634-z 35246111 PMC8895517

[12] World Health Organization. Global strategy to accelerate the elimination of cervical cancer as a public health problem and its associated goals and targets for the period 2020 – 2030. United Nations General Assembly; 2020. https://www.who.int/publications/i/item/9789240014107

[13] Hariprasad R, John A, Abdulkader RS. Challenges in the implementation of human papillomavirus self-sampling for cervical cancer screening in india: a systematic review. JCO Glob Oncol. 2023;9:e2200401. doi: 10.1200/GO.22.00401 36989463 PMC10281403

[14] Serrano B, Ibáñez R, Robles C, Peremiquel-Trillas P, de Sanjosé S, Bruni L. Worldwide use of HPV self-sampling for cervical cancer screening. Prev Med. 2022;154:106900. doi: 10.1016/j.ypmed.2021.106900 34861338

[15] Belinson JL, Du H, Yang B, Wu R, Belinson SE, Qu X, et al. Improved sensitivity of vaginal self-collection and high-risk human papillomavirus testing. Int J Cancer. 2012;130(8):1855–60. doi: 10.1002/ijc.26202 21630255

[16] Obiri-Yeboah D, Adu-Sarkodie Y, Djigma F, Hayfron-Benjamin A, Abdul L, Simpore J, et al. Self-collected vaginal sampling for the detection of genital human papillomavirus (HPV) using careHPV among Ghanaian women. BMC Womens Health. 2017;17(1):86. doi: 10.1186/s12905-017-0448-1 28950841 PMC5615631

[17] Fall NS, Tamalet C, Diagne N, Fenollar F, Raoult D, Sokhna C, et al. Feasibility, acceptability, and accuracy of vaginal self-sampling for screening human papillomavirus types in women from rural areas in Senegal. Am J Trop Med Hyg. 2019;100(6):1552–5. doi: 10.4269/ajtmh.19-0045 30994102 PMC6553900

[18] Bakiewicz A, Rasch V, Mwaiselage J, Linde DS. “The best thing is that you are doing it for yourself” - perspectives on acceptability and feasibility of HPV self-sampling among cervical cancer screening clients in Tanzania: a qualitative pilot study. BMC Womens Health. 2020;20(1):65. doi: 10.1186/s12905-020-00917-7 32234028 PMC7110708

[19] Andrew-Bassey UI, Oke DO, Okunlola MA, Morhason-Bello IO. Scoping review protocol on the perception and attitude of women on methods for collecting cervicovaginal samples for human papillomavirus testing in sub-Saharan Africa. BMJ Open. 2024;14(6):e085408. doi: 10.1136/bmjopen-2024-085408 38910004 PMC11328614

[20] Tricco AC, Lillie E, Zarin W, O’Brien KK, Colquhoun H, Levac D, et al. PRISMA extension for scoping reviews (PRISMA-ScR): checklist and explanation. Ann Intern Med. 2018;169(7):467–73. doi: 10.7326/m18-0850 30178033

[21] Morhason-Bello IO, Adebamowo CA. Epidemiology of uterine fibroid in black African women: a systematic scoping review. BMJ Open. 2022;12(8):e052053. doi: 10.1136/bmjopen-2021-052053 35922099 PMC9353014

[22] Saidu R, Moodley J, Tergas A, Momberg M, Boa R, Wright T, et al. South African women’s perspectives on self-sampling for cervical cancer screening: a mixed-methods study. S Afr Med J. 2018;109(1):47–52. doi: 10.7196/SAMJ.2018.v109i1.13278 30606304

[23] Kohler RE, Elliott T, Monare B, Moshashane N, Ramontshonyana K, Chatterjee P, et al. HPV self-sampling acceptability and preferences among women living with HIV in Botswana. Int J Gynaecol Obstet. 2019;147(3):332–8. doi: 10.1002/ijgo.12963 31489627 PMC6944206

[24] Berner A, Hassel SB, Tebeu P, Untiet S, Navarria I, Boulvain M. Women’s uncertainties over the reliability of the method are barriers to acceptance. 2013. 10.1097/LGT.0b013e31826b7b51 23422643

[25] Hartigan L, Cussen L, Meaney S, O’Donoghue K. Patients’ perception of privacy and confidentiality in the emergency department of a busy obstetric unit. BMC Health Serv Res. 2018;18(1):978. doi: 10.1186/s12913-018-3782-6 30563545 PMC6299575

[26] Federal Register. Rules and regulations, department of health and action. 2024:32976–33066.

[27] Yanikkerem E, Ozdemir M, Bingol H, Tatar A, Karadeniz G. Women’s attitudes and expectations regarding gynaecological examination. Midwifery. 2009;25(5):500–8. doi: 10.1016/j.midw.2007.08.006 18086509 PMC2801597

[28] Deesaen N, Sutantikorn K, Phonngoenchai P, Chaiyamahapruk S, Amatyakul P. Patients’ attitude and factors influencing the acceptance of medical students’ participation in pelvic examination. TAPS. 2022;7(1):87–97. doi: 10.29060/taps.2022-7-1/oa2519

[29] Nkwo PO, Chigbu CO, Nweze S, Okoro OS, Ajah LO. Presence of chaperones during pelvic examinations in southeast Nigeria: women’s opinions, attitude, and preferences. Niger J Clin Pract. 2013;16(4):458–61. doi: 10.4103/1119-3077.116889 23974739

[30] Skär L, Grankvist O, Söderberg S. Factors of importance for developing a trustful patient-professional relationship when women undergo a pelvic examination. Health Care Women Int. 2020;41(8):869–82. doi: 10.1080/07399332.2020.1716234 31951786

[31] Vega Crespo B, Neira VA, Ortíz S J, Maldonado-Rengel R, López D, Gómez A, et al. Evaluation of urine and vaginal self-sampling versus clinician-based sampling for cervical cancer screening: a field comparison of the acceptability of three sampling tests in a rural community of Cuenca, Ecuador. Healthcare (Basel). 2022;10(9):1614. doi: 10.3390/healthcare10091614 36141226 PMC9498379

[32] Devotta K, Vahabi M, Prakash V, Lofters AK. Implementation of a cervical cancer screening intervention for under- or never-screened women in Ontario, Canada: understanding the acceptability of HPV self-sampling. Curr Oncol. 2023;30(7):6786–804. doi: 10.3390/curroncol30070497 37504357 PMC10378307

[33] Fujita M, Nagashima K, Shimazu M, Suzuki M, Tauchi I, Sakuma M, et al. Acceptability of self-sampling human papillomavirus test for cervical cancer screening in Japan: a questionnaire survey in the ACCESS trial. PLoS One. 2023;18(6):e0286909. doi: 10.1371/journal.pone.0286909 37289798 PMC10249862

[34] Oneko O, Mahande MJ, Amour C, Pollie M, Smith C, Mboya IB, et al. Willingness to HPV self-sampling for cervical cancer screening and its predictors among women attending outpatient clinics in Meru District, Arusha Region, Northern Tanzania. Afr Health Sci. 2022;22(2):97–106. doi: 10.4314/ahs.v22i2.12 36407363 PMC9652623

[35] Omowhara B, Omosivie M, Soter A, Adekunbiola B. Effect of health education on the knowledge of cervical cancer, human papillomavirus and self-sampling among women in a low- resource setting. Euro J Med Heal Sci. 2022;4(3):145–51. doi: 10.24018/ejmed.2022.4.3.1316

[36] Hood RB, Turner AN, Huber-Krum S, Lancaster KE, Mwapasa V, Poindexter T, et al. For Human papillomavirus self-sampling, stated willingness does not correspond with subsequent uptake by rural malawian women. Sex Transm Dis. 2020;47(4):275–9. doi: 10.1097/OLQ.0000000000001119 32168286 PMC9808893

[37] Wu D, Lowry PB, Zhang D, Tao Y. patient trust in physicians matters-understanding the role of a mobile patient education system and patient-physician communication in improving patient adherence behavior: field study. J Med Internet Res. 2022;24(12):e42941. doi: 10.2196/42941 36538351 PMC9776535

[38] Zhu X, MacLaughlin KL, Fan C, Jacobson DJ, Jenkins GD, Jacobson RM, et al. Awareness of HPV testing and acceptability of self-sampling for cervical cancer screening among women in Minnesota. J Gen Intern Med. 2022;37(6):1565–8. doi: 10.1007/s11606-021-06854-x 33987791 PMC8118371

[39] Polman NJ, de Haan Y, Veldhuijzen NJ, Heideman DAM, de Vet HCW, Meijer CJLM, et al. Experience with HPV self-sampling and clinician-based sampling in women attending routine cervical screening in the Netherlands. Prev Med. 2019;125:5–11. doi: 10.1016/j.ypmed.2019.04.025 31054907

[40] Ezechi OC, Gab-Okafor CV, Ostergren PO, Odberg Pettersson K. Willingness and acceptability of cervical cancer screening among HIV positive Nigerian women. BMC Public Health. 2013;13:46. doi: 10.1186/1471-2458-13-46 23327453 PMC3567931

[41] Jenkins H, Jessiman WC, Hubbard G, O’Malley C. Exploring women’s experiences, views and understanding of vaginal examinations during intrapartum care: a meta-ethnographic synthesis. Midwifery. 2023;124:103746. doi: 10.1016/j.midw.2023.103746 37315454

[42] Dadi TL, Tegene Y, Vollebregt N, Medhin G, Spigt M. The importance of self-management for better treatment outcomes for HIV patients in a low-income setting: perspectives of HIV experts and service providers. AIDS Res Ther. 2024;21(1):28. doi: 10.1186/s12981-024-00612-9 38704594 PMC11070098

[43] Waldron EM, Burnett-Zeigler I, Wee V, Ng YW, Koenig LJ, Pederson AB, et al. Mental health in women living with HIV: the unique and unmet needs. J Int Assoc Provid AIDS Care. 2021;20:2325958220985665. doi: 10.1177/2325958220985665 33472517 PMC7829520

[44] Manhas C. Self-esteem and quality of life of people living with HIV/AIDS. J Health Psychol. 2014;19(11):1471–9. doi: 10.1177/1359105313493812 23864076

[45] Lozar T, Nagvekar R, Rohrer C, Dube Mandishora RS, Ivanus U, Fitzpatrick MB. Cervical cancer screening postpandemic: self-sampling opportunities to accelerate the elimination of cervical cancer. Int J Womens Health. 2021;13:841–59. doi: 10.2147/IJWH.S288376 34566436 PMC8458024

[46] Review S. HHS Public Access. 2021. doi: 10.1016/j.ypmed.2019.105953.COST-EFFECTIVENESS

