# Supplementary material for: Scoping review on the perceptions and attitude of women on methods for collecting cervicovaginal samples for Human Papillomavirus testing in Sub-Saharan Africa
Source: PLOS Glob Public Health. 2025 May 23;5(5):e0004641. doi: 10.1371/journal.pgph.0004641 (PMC12101692; doi:10.1371/journal.pgph.0004641)
Supplement: S1 Table — (DOCX) [file pgph.0004641.s001.docx]

Table 1: Table Showing Eligibility Criteria for the Review using PICO Format

| **Categories** | **Inclusion Criteria** | **Exclusion Criteria** |
| --- | --- | --- |
| **Participants** | Women between the ages 18-65 years that have been previously screened for cervical cancer | Transgendered women without a cervix, and women that have never screened for cervical cancer |
| **Intervention** | Cervical cancer screening with samples collected either by oneself or by a health care provider or both | Samples collected from the cervix for other purposes other than cervical cancer screening |
| **Comparator** | Self-sampling; Provider sampling | Provider sampling only; Self-sampling only |
| **Outcome** | Perception, attitude, perspective, and/or acceptability | - |
