# Supplementary material for: Scoping review on the perceptions and attitude of women on methods for collecting cervicovaginal samples for Human Papillomavirus testing in Sub-Saharan Africa
Source: PLOS Glob Public Health. 2025 May 23;5(5):e0004641. doi: 10.1371/journal.pgph.0004641 (PMC12101692; doi:10.1371/journal.pgph.0004641)
Supplement: S2 Table — (DOCX) [file pgph.0004641.s002.docx]

**TABLE 2: Description of Included Studies, Method of participant’s Recruitment, and Type of Cervical Screening Conducted**

| **S/N** | **Author (s)/Year/Country** | **Study Population/ Sample Size** | **Method of Recruitment** | **Type of Screening** | **Procedure for sample collection** |
| --- | --- | --- | --- | --- | --- |
| 1 | ^1^Saidu et al. | Women ages 30-65 years/822 questionnaires administered and 41 FGD’s | Women referred to a colposcopy clinic and a primary care site in Cape Town, SA were enrolled consecutively from February 2015 to May 2016. Equal numbers of HIV-positive and HIV-negative women were recruited. | Self-sampling (SS) for HPV testing followed by VIA and Colposcopy, appropriate histology specimens by a doctor. | All the participants self-collected a vaginal sample in a private room following a verbal explanation on how to collect the sample by a community health worker (CHW). |
| 2 | ^2^Sormani et al. | Women 30-49 years/ 2201 | Women referred to a colposcopy clinic and a primary care site in Cape Town, SA, from February 2015 to May 2016. Equal numbers of HIV-positive and HIV-negative women were recruited. No account for non-response rate. | SS for HPV testing, VIA & VILI conducted for positives, then treated by thermal ablation or loop electrosurgical excision of the transformation zone (LEETZ) | Women received instructions and a support guide that provided detailed visual information about the procedure. |
| 3 | ^3^Obiri-Yeboah et al. | Women aged 18 years and above/194 | Women attending the general medical outpatient and HIV clinics were recruited. A systematic random sampling of every fifth woman were selected from the list of daily attendants | SS for HPV testing and HCP samples for HPV testing | Recruited participants at the clinic were instructed on how to obtain Self collect vaginal samples using the careHPV brush and transport medium. Speculum examination was also conducted by the using a similar brush and transport media. |

^1^Saidu et al. 2019/ South Africa, ^2^Sormani et al. 2021/Cameroon, ^3^Obiri-Yeboah et al. 2017/ Ghana, SS, Self-Sampling; HCP, Healthcare Provider

**TABLE 2: (continued)**

| **S/N** | **Author (s)/Year/Country** | **Study Population/ Sample Size** | **Method of Recruitment** | **Type of Screening** | **Procedure for sample collection** |
| --- | --- | --- | --- | --- | --- |
| 4 | ^4^Kohler et al. | WLWH aged 25 years and above/ 104 | WLWH attending an HIV clinic for routine healthcare in Gaborone were recruited between March and April 2017. | SS for HPV testing and speculum examination by a clinician afterwards | Verbal description of how to use SS kit and distributed pictorial instructions. The cephid patient-collected vaginal swab instructions were adapted. |
| 5 | ^5^Bakiewicz et al. | Women aged 25–60 years attended a patient-initiated screening/21 | A non-random (purposive) sampling technique was used | SS for HPV, HCP sampling, gynaecologic examination and VIA. | Written instruction guide with illustrations of how to collect the self-sample as well as an oral instruction provided by a nurse to collect self-sample |
| 6 | ^6^Berner et al. | Women aged 25-65 years/ 243 | Recruitment of women attending routine cervical screening through convenience sampling | SS for HPV testing, HCP sampling and cytology | Written and oral instructions were given to self-sample unsupervised |

^4^Kohler et al. 2019/Botswana, ^5^Bakiewicz et al., 2020/Tanzania, ^6^Berner et al. 2013/ Cameroon; SS, Self-Sampling; HCP, Healthcare Provider
