# Supplementary material for: Scoping review on the perceptions and attitude of women on methods for collecting cervicovaginal samples for Human Papillomavirus testing in Sub-Saharan Africa
Source: PLOS Glob Public Health. 2025 May 23;5(5):e0004641. doi: 10.1371/journal.pgph.0004641 (PMC12101692; doi:10.1371/journal.pgph.0004641)
Supplement: S3 Table — (DOCX) [file pgph.0004641.s003.docx]

**TABLE 3: Selected Studies on Perception and Attitude of Self-Sampling in Sub-Saharan Africa Reporting Key Findings**

| **S/N** | **Author (s)/Year/Country** | **Aim: Perception/Attitude** | **Measurement for Perception (Quantitative/Qualitative/Mixed)** | **Assessment for Perception (Descriptive/Multivariate/ Test of Association)** | **Outcome Measure** | **Key Findings** |
| --- | --- | --- | --- | --- | --- | --- |
| 1 | ^1^Saidu et al. | To explore women’s perceptions and acceptance of self-collection of samples for cervical screening and their willingness to do so, in a low-resource setting in South Africa (SA). | Mixed: quantitative assessment with a Likert scale and qualitative from the FGD. The questionnaire was self-administered | Mean/SD/ p-value; thematic analysis for FDG | Perceptions and beliefs surrounding SS | Positive attitude to SS was 93.6%. Positive perception to SS was 89.4%. FGD participants found SS easier, more comfortable, and less embarrassing than HCP sampling. Willingness to SS but expressed concerns regarding the quality of the specimen and the financial implications of returning to the clinic with it. |
| 2 | ^2^Sormani et al. | To assess factors associated with women's preferences related to self-HPV and perceptions around Self-HPV | Quantitative data using interviewer-administered questionnaire | mean(SD), frequency(percentage), chi-square, logistic regression | preference for clinician-sampled versus self-HPV | Comfortability was reported in 98.3% for SS and 96.9% during clinician sampling. Confidence: 99.3% SS vs 98.6% clinician sampling. Clinician expertise made women to rely more on HCP method compared to SS (76.1%). |
| 3 | ^3^Obiri-Yeboah et al. | To determine the acceptability, feasibility and performance of alternative self-collected vaginal samples for HPV detection among Ghanaian women. | Quantitative data with interviewer-administered questionnaire | Frequency/Percentage, kappa for concordance | Acceptability, concordance | 76.3% easier felt SS was than in 77.9% for HCP sampling. 57.7% would prefer SC over HCP sampling and 61.9% felt SC would increase their likelihood to access cervical cancer screening |
| 4 | ^4^Kohler et al. | To assess the acceptability and preferences of HPV screening with SS and mobile phone results delivery among women living with HIV. | Quantitative data with interviewer-administered questionnaire | Frequency/Percentage, chi-square test | Acceptability of SS | 90% found SS to be easy and comfortable. 95% expressed willingness for SS again (95%), 19% preferred SS over HCP sampling. A high number trust their clinicians & had low confidence in being able to sample correctly. |

^1^Saidu et al. 2019/ South Africa, ^2^Sormani et al. 2021/Cameroon, ^3^Obiri-Yeboah et al. 2017/ Ghana, ^4^Kohler et al. 2019/Botswana; SS, Self-Sampling; HCP, Healthcare Provider

**TABLE 3: (continued)**

| **S/N** | **Author (s)/Year/Country** | **Aim: Perception/Attitude** | **Measurement for Perception (Quantitative/Qualitative/Mixed)** | **Assessment for Perception (Descriptive/Multivariate/ Test of Association)** | **Outcome Measure** | **Key Findings** |
| --- | --- | --- | --- | --- | --- | --- |
| 5 | ^5^Bakiewicz et al. | To investigate the feasibility and acceptability of HPV SS among Tanzanian women who attended a patient-initiated cc screening compared to provider-based HPV sampling. | Qualitative data with IDI | Thematic analysis | Feasibility and acceptability | Most women perceived SS as easy and comfortable though few experienced bleeding and pain. |
| 6 | ^6^Berner et al. | To assess acceptability and preference for self-collected HPV tests compared with traditional physician-sampled Pap tests in a low-resource country | Quantitative data using a self-administered questionnaire | mean (SD), frequency (percentage), Mann-Whitney U-test, McNemar, binomial, chi-square, multivariate logistic regression | Acceptability and preference of SS over physician sampling | The acceptability score for self-HPV was 9.20 versus 11.80 for physician sampling (p<0.001). Preference was lower for self-HPV than physician sampling (29% vs 62%; p<0.001). |

^5^Bakiewicz et al., 2020/Tanzania, ^6^Berner et al. 2013/ Cameroon; SS, Self-Sampling; HCP, Healthcare Provider
