# Supplementary material for: Scoping review on the perceptions and attitude of women on methods for collecting cervicovaginal samples for Human Papillomavirus testing in Sub-Saharan Africa
Source: PLOS Glob Public Health. 2025 May 23;5(5):e0004641. doi: 10.1371/journal.pgph.0004641 (PMC12101692; doi:10.1371/journal.pgph.0004641)
Supplement: S1 Data — (DOCX) [file pgph.0004641.s004.docx]

**SCOPING REVIEW SEARCH STRATEGY**

**Title: A scoping review protocol on the perception and attitude of women on methods for collecting cervico-vaginal samples for Human Papillomavirus testing in Sub-Saharan Africa**

**Intended Databases:** Cochrane Library, PUBMED, African Journal Online & Google Scholar

**Keywords:** HPV/ Human papillomavirus/ papillomavirus, cervical cancer/ cervical squamous/ adenocarcinoma, Cervico-vaginal/cervico-vaginal samples, sample collection/ sample taking, cervical cancer screening, perception/view, attitude, and SSA using sub-regions within SSA (West Africa OR East Africa OR Central Africa OR Southern Africa), and by specific country names.

Filter: Publications between 2013 and 2023

| **Variable** | **Cochrane** | **Pubmed** | **AJOL** | **Google Scholar** | **Manual Search** |
| --- | --- | --- | --- | --- | --- |
| Date of 1^st^ search | 02/01/2024 | 29/12/2023 | 30/12/2023 | 30/12/2023 | 30/12/2023 |
| Date of last search | 23/03/2024 | 20/03/2024 | 24/03/2024 | 26/03/2024 | 28/03/2024 |
| #1: Human papillomavirus and others synonyms; HPV, HPV testing, papillomavirus testing | (Human papillomavirus samples): ti,ab,kw OR (HPV testing): ti,ab,kw OR (papillomavirus testing): ti,ab,kw OR (HPV) | (((((Human papillomavirus samples [Title/Abstract]) OR (HPV testing [Title/Abstract])) OR (papillomavirus testing [Title/Abstract])) OR (HPV [Title/Abstract])) | Humanpapilloma virus testing | Humanpapilloma virus testing | Humanpapilloma virus testing |
| #2: Cervico-vaginal samples and synonyms such as cervical samples, samples from the cervix | (Cervico-vaginal samples): ti,ab,kw OR ( self-sample): ti,ab,kw OR (cervical samples): ti,ab,kw OR ( samples from the cervix): ti,ab,kw | (Cervico-vaginal samples) [Title/Abstract]) OR (self-sample) [Title/Abstract]) OR (cervical samples) [Title/Abstract]) OR ( samples from the cervix) [Title/Abstract])) | cervico-vaginal samples OR self-sample OR sample collection | cervico-vaginal samples OR self-sample OR sample collection | cervico-vaginal samples OR self-sample OR sample collection |
| #3: Cervical cancer screening approaches and synonyms such as squamous cell carcinoma screening, cervical adenosarcoma screening, cervical alveolar soft part sarcoma screening, cervical angiosarcoma screening | (cervical cancer screening):ti,ab,kw OR (squamous cell carcinoma screening):ti,ab,kw OR (cervical adenosarcoma screening):ti,ab,kw OR (cervical alveolar soft part sarcoma screening):ti,ab,kw OR (cervical angiosarcoma screening):ti,ab,kw | (cervical cancer screening) [Title/Abstract]) OR (squamous cell carcinoma screening) [Title/Abstract]) OR (cervical adenosarcoma screening) [Title/Abstract]) OR (cervical alveolar soft part sarcoma screening) [Title/Abstract]) OR (cervical angiosarcoma screening) [Title/Abstract])) | cervical cancer screening | cervical cancer screening | cervical cancer screening |
| #4: Perception, attitude and synonyms such as views, perspective | ("perception"):ti,ab,kw OR (attitude):ti,ab,kw OR (views):ti,ab,kw OR (perspective):ti,ab,kw | ("perception") [Title/Abstract]) OR (attitude) [Title/Abstract]) OR (views) [Title/Abstract]) OR (perspective) [Title/Abstract]) | perception OR attitude OR views OR perspective | perception OR attitude OR views OR perspective | perception OR attitude OR views OR perspective |
| #5: Women and synonyms such as Woman, elderly female, female, lady | (Women):ti,ab,kw OR (Woman):ti,ab,kw OR (Elderly female):ti,ab,kw OR (Female): ti,ab,kw OR (Lady) | (Women) [Title/Abstract]) OR (Woman) [Title/Abstract]) OR (Elderly female) [Title/Abstract]) OR (Female) [Title/Abstract]) OR (Lady) | women | women | women |
| #6: Sub-Saharan Africa and regions in Sub-Saharan Africa such as Central Africa, Southern Africa, West Africa, East Africa | ("sub-Saharan Africa"):ti,ab,kw OR (Central Africa):ti,ab,kw OR (Southern Africa):ti,ab,kw OR (West Africa):ti,ab,kw OR (East Africa):ti,ab,kw | ("sub-Saharan Africa") [Title/Abstract]) OR (Central Africa) [Title/Abstract]) OR (Southern Africa) [Title/Abstract]) OR (West Africa) [Title/Abstract]) OR (East Africa) [Title/Abstract]) | subsaharan Africa | subsaharan Africa | subsaharan Africa |
| #7: #1 AND #2 AND #3 AND #4 AND #5 AND #6 | **3** | **18** | **58** | **53** | **5** |
